# Robust Scale Adaptive Tracking by Combining Correlation Filters with Sequential Monte Carlo

**DOI:** 10.3390/s17030512

**Published:** 2017-03-04

**Authors:** Junkai Ma, Haibo Luo, Bin Hui, Zheng Chang

**Affiliations:** 1 Shenyang Institute of Automation, Chinese Academy of Sciences, Shenyang 110016, China; luohb@sia.cn (H.L.); huibin@sia.cn (B.H.); changzheng@sia.cn (Z.C.); 2 Key Laboratory of Opto-Electronic Information Processing, Chinese Academy of Sciences, Shenyang 110016, China; 3 University of Chinese Academy of Sciences, Beijing 100049, China

**Keywords:** target tracking, sequential Monte Carlo framework, correlation filter, scale estimation, occlusion

## Abstract

A robust and efficient object tracking algorithm is required in a variety of computer vision applications. Although various modern trackers have impressive performance, some challenges such as occlusion and target scale variation are still intractable, especially in the complex scenarios. This paper proposes a robust scale adaptive tracking algorithm to predict target scale by a sequential Monte Carlo method and determine the target location by the correlation filter simultaneously. By analyzing the response map of the target region, the completeness of the target can be measured by the peak-to-sidelobe rate (PSR), i.e., the lower the PSR, the more likely the target is being occluded. A strict template update strategy is designed to accommodate the appearance change and avoid template corruption. If the occlusion occurs, a retained scheme is allowed and the tracker refrains from drifting away. Additionally, the feature integration is incorporated to guarantee the robustness of the proposed approach. The experimental results show that our method outperforms other state-of-the-art trackers in terms of both the distance precision and overlap precision on the publicly available TB-50 dataset.

## 1. Introduction

Visual object tracking plays an important role in computer vision. It is a basic component within a variety of applications including surveillance, human–computer interaction, action recognition and robotics, etc. The performance of these applications depends on the accuracy of the object tracking algorithms. Though numerous precise and steady algorithms were proposed in recent years, there still exist challenges in object tracking, which are mainly caused by illumination changes, partial occlusion, background clutter and nonrigid deformation in natural scenes.

To address the challenges that appeared in the tracking mission, lots of researchers developed some sophisticated approaches [1,2,3,4,5,6,7]. The popular tracking algorithm can be categorized into generative and discriminative methods. The generative method seeks to consider tracking as a problem of finding the maximal-similarity region to the target. The target is represented as a template [8] or parameter model in feature space [9,10]. The similarity is measured in feature space or a low-dimensional subspace to describe the target and incrementally learn the subspace to adapt to appearance changes during tracking. Zhong et al. represented the target as a sparse dictionary within a particle filter framework in [2]. The discriminative method formulates the tracking problem as a binary classification task whose goal is to discriminate the target from the background [1,3,11,12,13]. Usually, this type of approach consists of three stages: (1) using the classifier to distinguish the target from background; (2) sampling some positive and negative samples according to how much the corresponding region includes the target object; and (3) updating the classifier using the labeled samples. It proceeds within the above three stages iteratively upon every frame. The performance of discriminative tracking is largely dependent on the specific binary classifier.

To improve the computational speed, a correlation filter based tracking method has been widely researched [4,14,15]. The correlation filter has been applied in the signal processing field for several decades. The correlation between these two signals can be seen as their similarity. The convolution operation in the time domain can be effectively computed in the Fourier domain by element-wise multiplication. This property can be used to reduce the computational burdens. The extension of correlation filters to tracking achieves high frame-rates. Unfortunately, the size of the template is fixed, which limits its applications, especially in the context of target scale variation.

The scale variation is one of the main challenges in the tracking. It influences the tracking performance in two respects. Firstly, the target features exhibit multi-level difference when the target scale changes. It triggers tracking inaccuracy while searching for the candidate in a fixed scale space. Secondly, the fixed scale further contributes to model update inaccuracy (i.e., if the tracking bounding box is bigger than the target, the background information is usually contained; on the contrary, if the tracking bounding box is smaller than the target, it suffers from the loss of target information). Thus, the scale variation can degrade the representational ability of the model.

Additionally, occlusion is another tough issue which also impacts the tracker’s performance. When the occlusion occurs, the desired target region similarity degrades and cluttered background distracts the template to match with other mistaken regions. Both of these two factors jointly result in tracking failure. To make it worse, the template is updated with the false information, which further brings unsatisfactory templates to the subsequent frames. Thus, occlusion is supposed to be paid attention to while designing the accurate and robust tracking algorithms.

In this paper, we propose a robust scale adaptive tracking algorithm based on the correlation filter. The main contributions of our work are listed below:

- We define the scale variable of the target to measure the scale variation during the tracking. Afterwards, we design a method to estimate the scale variable using the Sequential Monte Carlo Framework.
- We analyze the correlation response map under the circumstances of various levels of target occlusion. Furthermore, the peak-to-sidelobe rate (PSR) is employed to measure the degree of occlusion. It has been verified on a large number of video sequences.
- A model update strategy is designed according to the stability of the target region during tracking. It’s remarkable that this strategy gracefully strikes the balance between the target appearance changes and the model drifts.

The reminder of paper is organized as follows. Previous related works are reviewed briefly in Section 2. The correlation filter in tracking is described in Section 3. The details of our work are shown in Section 4. The experiments on several challenging sequences are preformed and analysis is given in Section 5. Section 6 concludes the whole paper.

## 2. Related Work

Tracking-by-detection trackers achieve high performance in currently published literature. In this section, we briefly review some of them that are closely related to our work. More detailed review of this kind of work is described in these papers [16,17].

The tracking-by-detection algorithms usually employ a classifier to discriminate the target and background. Babenko et al. [11] proposed a tracking method named MIL, which trains the classifier online through bags of samples instead of the labeled individual instance set. The latter relies heavily on the labeled sample precision, i.e., a slight labeling mistake can lead to severe degradation of the classifier. In contrast, the former one, however, can avoid this limitation, which, in turn, makes the classifier more robust. Kalal et al. [1] proposed the TLD algorithm. This method decomposes a long-term tracker into three components: tracking, learning, and detection. In each frame, the tracker follows the target; the detector localizes the regions similar enough to the target and corrects the tracking results; and the learner evaluates and updates the detector to improve its performance. Hare et al. [3] proposed the Struck tracker using the structured output Support Vector Machine (SVM). It utilizes the structure information to train the classifier to ensure the structure output accuracy. Zhang et al. [13] leveraged compressive sensing theory that projects the high-dimension features into a low-dimension space. The high-dimension features contain rich information of the target and the random projection can preserve the structure of the image feature space. Both of these factors guarantee feature discrimination in low-dimensional space. Then, a naive Bayes classifier is used to discriminate the target from the background in the compressed domain.

Many algorithms are designed to handle the target scale variation in tracking. SCM [2] and L1APG [18] used particle filter framework to estimate the target state. The target scale is one of dimensions in that state space. CMT [5] used a set of keypoints to represent the target. The target scale is measured by computing the geometry relationship between the pairwise keypoints.

A variety of trackers [4,6,14,15] were proposed based on correlation filter, which has been researched for several decades in signal processing [19]. The Minimum Output Sum of Squared Error (MOSSE) tracker [15] trains the filter coefficients by minimizing the sum of squared error between the filter response and the desired response. By transferring the model into the Fourier domain, the matrix algebra can be solved by element-wise operation. By means of this, MOSSE can run at an impressive speed. The Circulant Structure tracker with Kernels (CSK) [14] extended the MOSSE by exploiting the redundancy of the sampling subwindows. This solved the expensive computation of dense sampling through a cyclically shifted sampling method. This study also proved that the kernel matrix of the samples also has circulant structure. Therefore, a non-linear kernel can be introduced into the tracker straightforwardly. The CSK is the preliminary version of the Kernelized Correlation Filter (KCF). To remedy the drawback of CSK, which is limited to a single channel feature, KCF [4] can deal with multiple channel features that make the tracking results more accurate.

The closest works to ours are the Discriminative Scale Space Tracker (DSST) [6] and Scale Adaptive with Multiple Features tracker (SAMF) [20]. DSST employs the correlation filter as the basic tracking and introduces a one-dimension filter to determine the target scale. SAMF used a fixed scaling pool to sample the candidates at different sizes. On top of that, our work treats the target scale prediction as estimation of a one-dimensional signal through the preview observations, which has been solved by a probability technique. Our method can tackle the scale variance more simply and efficiently.

## 3. Kernelized Correlation Filter

The Convolution Theorem states that the convolution of two signals in the time domain can be computed by element-wise multiplication in the frequency domain, which is much more efficient. This property can be straightforwardly applied to two-dimensional images. The similarity of two image patches can be measured by the correlation between them. In the correlation filter based tracker, image patches are transferred into a frequency domain by discrete Fourier transformation (DFT) and the correlation is calculated in the frequency domain. Then, the spatial correlation response map can be obtained through inverse DFT. In this section, we briefly introduce the KCF [4], which is the basis of our tracker.

The goal of the KCF is to learn a function f(x)=〈w,x〉 that minimizes the squared error over samples $x_{i}$ and their regression targets $y_{i}$,

(1) $$\min_{w}\sum_{i}{\left(f\left(x_{i}\right)-y_{i}\right)}^{2}+\lambda {∥w∥}^{2},$$

where *λ* is a regularization parameter. The closed-form solution is given by:

(2) $$w={\left(X^{T}X+\lambda I\right)}^{-1}X^{T}y,$$

where *X* is the data matrix that has one sample $x_{i}$ per row, I is an identity matrix, and y is a vector containing the regression target $y_{i}$ corresponding to each sample $x_{i}$.

The KCF trains this model by an image patch x of size W×H that contains the target object. Each training sample $x_{w,h}$ is obtained by cyclically shifting x with *w* pixels horizontally and *h* pixels vertically, where (w,h)∈{0,1,...,W−1}×{0,1,2,...,H−1}. The regression target y simply follows a 2D Gaussian function. Solving Equation (2) is very time consuming because it contains matrix operation, especially matrix inversion. By converting it into the frequency domain, Equation (2) can be solved more efficiently. Utilizing the property of the circulant matrix and the DFT, the solution of the Equation (1) is given in the frequency domain as:

(3) $$\hat{w}=\frac{{\hat{x}}^{*}⊙\hat{y}}{{\hat{x}}^{*}⊙\hat{x}+\lambda },$$

where ⊙ indicates the element-wise product, $\hat{x}$ denotes the DFT of the x, and ${\hat{x}}^{*}$ means the complex-conjugate of $\hat{x}$. Using the dual technique, the model parameter w can be rewritten in dual space: $w=\sum_{i}\alpha_{i}\varphi \left(x_{i}\right)$, where the $\varphi (x_{i})$ means mapping the sample $x_{i}$ into a feature space. The regression function of a image patch z can be expressed as:

(4) $$f\left(z\right)=\langle w,\varphi \left(z\right)\rangle =\sum_{i=1}^{n}\alpha_{i}\langle \varphi \left(z\right),\varphi \left(x_{i}\right)\rangle .$$

The inner product can be rewritten as: $\langle \varphi \left(x\right),\varphi \left(x^{\prime }\right)\rangle =\kappa \left(x,x^{\prime }\right)$, where κ(·,·) is a kernel function. The kernelized version solution can be expressed as:

(5) $$\alpha ={\left(K+\lambda I\right)}^{-1}y,$$

where *K* is a kernel matrix with element $K_{ij}=\kappa \left(x_{i},x_{j}\right)$. The solution is given in the frequency domain as:

(6) $$\hat{\alpha }=\frac{\hat{y}}{{\hat{k}}^{xx}+\lambda },$$

where $k^{xx}$ is the first row of the kernel matrix *K*, and x is the target appearance model. In a new frame, an image patch *z* with the same size of *x* is cropped out, and the response map is calculated by:

(7) $$\hat{f}\left(z\right)={\hat{k}}^{xz}⊙\hat{\alpha },$$

where ${\hat{k}}^{xz}=\kappa \left(z,\hat{x}\right)$. The location of the maximal element in the spatial response map f(z) indicates the patch that is the most similar to the target appearance.

## 4. Proposed Method

The drawback of the KCF is that the size of the tracking bounding box is fixed. This makes the tracker inaccurate in the context of target scale variance. To overcome this nontrivial problem, we intentionally design a robust tracking algorithm to remedy this limitation. We use the KCF with a integrated feature as the basic tracker and employ the Sequential Monte Carlo Framework to predict the scale of the target. We also utilize the peak-to-sidelobe ratio to measure the completeness of the target, which is also considered as a measurement of the occlusion degree. Based on this measurement, a template update strategy and a retained scheme are designed to cope with the target appearance change and the occlusion, respectively. The details of our approach are given as follows.

### 4.1. Scale Estimation with Sequential Monte Carlo

The KCF can efficiently locate the target in each frame, but the size of the model coefficient $\hat{\alpha }$ and the target appearance x are fixed. It can not handle the scale variation of the target. When the scale change occurs, the tracker is prone to drift. To cope with this challenge, we explore the Sequential Monte Carlo Framework to estimate the scale of the target.

In order to deal with the scale change of target in tracking, we define a scale variable $s_{t}$ to indicate the size of the target in the *t*-th frame. As the general case, the target is represented by a bounding box $x_{t}=\left[x_{t},y_{t},w_{t},h_{t}\right]$ in the *t*-th frame. The scale variable of the target in the *t*-th frame is defined as $s_{t}=\sqrt{w_{t}*h_{t}/w_{1}*h_{1}}$. By this definition, the estimation of the target scale can be regarded as the estimation of a one-dimensional variable by the observation set $O^{\left(t\right)}=\left(o_{1},o_{2},...,o_{t}\right)$, which means the frames of the sequences up to the *t*-th frame. Given the available observations $O^{t-1}$, the distribution of scale variable $s_{t}$ is predicted as:

(8) $$p\left(s_{t}|O^{t-1}\right)=\int p\left(s_{t}|s_{t-1}\right)p\left(s_{t-1}|O^{t-1}\right)ds_{t-1},$$

where $p(s_{t}|s_{t-1})$ is the transition density function and $p(s_{t-1}|O^{t-1})$ is the state density function. When observation $o^{t}$ is given in the *t*-th frame, the posterior probability can be calculated recursively by the Bayes rule:

(9) $$p\left(s_{t}|O^{t}\right)=\frac{p\left(o_{t}|s_{t}\right)p\left(s_{t}|O^{t-1}\right)}{p(o_{t}|O^{t})},$$

where $p(o_{t}|s_{t})$ is the observation likelihood. The stated variable $s_{t}$ is modeled by a Gaussian distribution around the $s_{t-1}$:

(10) $$p\left(s_{t}|s_{t-1}\right)=N\left(s_{t};s_{t-1},\sigma^{2}\right).$$

It means that the state of the target distributes around the state in the previous frame with a variance *σ*. From Equation (8) to (9), it is obvious that maximizing the posterior probability is equivalent to maximizing the observation likelihood. The KCF can calculate the response score of the image patch with the same size of the template *x* efficiently and generate a response map $f_{s_{t}}\left(z\right)$ in the specific scale $s_{t}$. To estimate the scale of the target, we should define the observation mode as:

(11) $$p\left(o_{t}|s_{t}\right)=\max f_{s_{t}}\left(z\right).$$

When a new observation (frame) comes, image patches were captured in several scale spaces based on the scale variable $s_{t}$. $s_{t}$ is sampled by the Equation (10). After these image patches are resized into the KCF model size, response maps of them are calculated efficiently by the correlation filter. The maximal value of every response map is defined as the the observation likelihood of the corresponding scale. Through the Equations (8)–(10), the target scale variable $s_{t}$ can be predicted recursively in every frame during the tracking.

### 4.2. Occlusion Measurement

**Figure 1:**
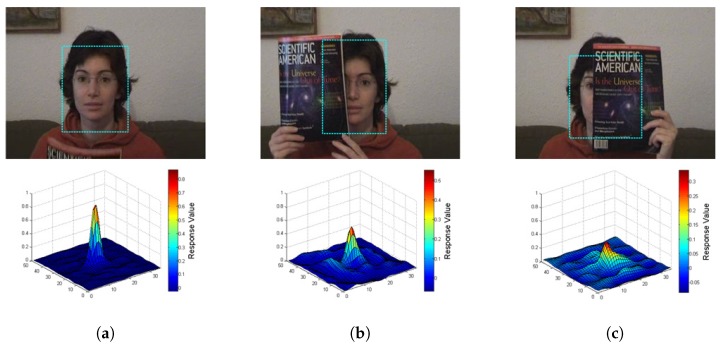
Illustration of the occlusion caused by a dissimilar object in the *faceocc1* sequence. The *z*-axis range of the response map is the same, in order to compare the differences in the peak values in different occlusion states. (**a**) Non-occlusion target and its response map; (**b**) Slight occlusion target and its response map; (**c**) Heave occlusion target and its response map.

**Figure 2:**
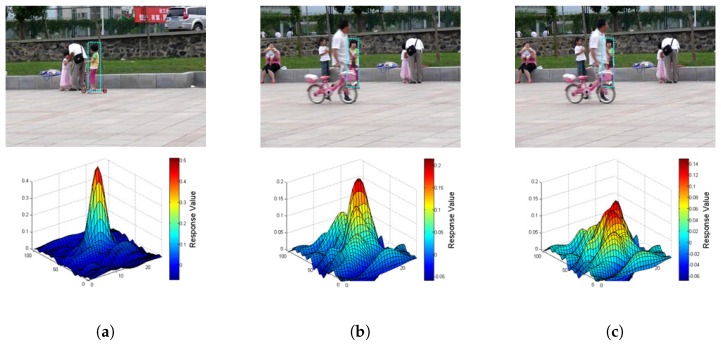
Illustration of the occlusion caused by a similar object in the *girl2* sequence. The *z*-axis range of the response map is different, in order to clarify the value of the sidelobes in different occlusion states. (**a**) Non-occlusion target and its response map; (**b**) Slight occlusion target and its response map; (**c**) Heave occlusion target and its response map.

Occlusion is also a challenging issue in target tracking. The preliminary step in handling occlusion is to measure how much the target has been occluded. In this section, we analyze the shape of the response map to determine the degree of target occlusion. Three common target states—non-occlusion, slight occlusion, and heave occlusion—are shown in Figure 1 and Figure 2. The subfigures in the upper row are the original frames in the sequence; the counterparts in the lower row are the response map of the corresponding target region, respectively. From the Figure 1, it is obvious that the more complete the target is, the more similar it is compared with the template. Therefore, the response value of the non-occlusion state is higher than that of the occlusion state. We can conclude that the peak value of the response map indicates the completeness of the target. Figure 2 shows a more complex circumstance when the target is occluded by a similar object. When a similar object occludes the target, the response map will have multiple peaks. This means that the sidelobes have a very high response value in addition to the main lobe. Occlusions caused by similar and dissimilar objects are both considered to be in an unstable state in tracking. Inspired by [15], we use the peak-to-sidelobe ratio (PSR) to measure how stable the target region is. PSR of a response map is defined as:

(12) $$PSR\left(x\right)=\frac{max(x)-\mu (x)}{\sigma (x)},$$

where μ(x) is the mean of the response map *x*, and σ(x) is the standard deviation of *x*.

**Figure 3:**
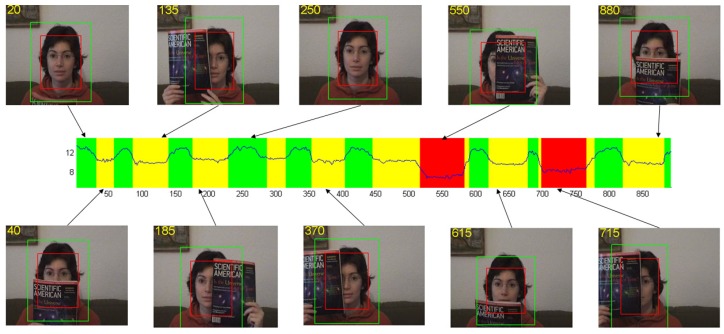
PSR (peak-to-sidelobe rate) plot, where the curve plots the PSR of every frame in *faceocc1* sequence. The area enclosed by the red rectangle is the target area, and the one enclosed by the green rectangle is the whole candidate area.

According to the PSR, the occlusion degree of the target can be successfully measured in the tracking. To verify this measurement, we plot the response map PSR in a whole sequence in Figure 3. The curve which is plotted in the middle of the figure shows the PSR of every frame in the *faceocc1* sequence. The background color is marked depending on the PSR to indicate the degree of occlusion. Green means that the target is normal without occlusion in these frames. Yellow means that the target is partially occluded, and red means that the target is almost totally occluded. The frames corresponding to these scenarios are shown above and below the curve. The target state in these frames is consistent with the measurement of PSR.

A threshold $T_{d}$ is set to handle the occlusion. When PSR is smaller than $T_{d}$, it means that the object is heavily occluded. In this case, the tracking result is unreliable. In order to avoid tracker drifting, we apply a retained strategy in this situation. This strategy employed a simple but valid assumption that the target will remain in the same position until the target reappears or the occlusion is removed. This assumption is valid based on the following fact. In most tracking videos, the occlusion can be divided into two cases. One is that another object moves and covers the target, and the other is that the target moves to the back of some static object in the background. In the first case, the occlusion is caused by a moving object so it is obvious that the target will stay in the same position when the covering moves away. In the second case, the target always reappears near the static object in the background. Thus, we keep the search region in the same position, making it is easy for capturing the target again, and this benefits from the search region of the correlation filter being 2.5 × 2.5 times the size of the target. Therefore, our tracker retains the scale and the position of the target in the previous frame as the tracking result in the current frame. Through this strategy, when the target reappears, our tracker can quickly find out the new location of the target.

### 4.3. Update Strategy

The appearance of the target changes during the tracking by rotation, deformation, etc. Therefore, the target template should be updated during the tracking to get a robust performance. If the target template is updated too frequently, the template is prone to be corrupted by noise. On the contrary, if the target template is updated too slowly, the template can not capture the normal appearance change of the target. A suitable update scheme is crucial for a tracker.

Using the target state measurement described above, the lower the PSR, the more serious the target is occluded, and we can easily design a reliable update scheme. For each frame, we firstly calculate the PSR of the target region response map. A threshold $T_{u}$ is set to determine whether the template needs to be updated or not. $PSR\left(x\right)<T_{u}$ means that the target is partially occluded. It will corrupt the template that updates with the tracking result in this frame. Therefore, we only update the template in these frames where the PSR of the response map is higher than $T_{u}$.

When updating is needed, the model coefficients *α* and the template appearance $x^{t}$ is updated following the formulas in KCF. When the new target bounding box $x^{\prime }$ is captured by the tracker, the coefficients of the model are updated by:

(13) $$\begin{array}{cc} {\hat{\alpha }}^{t} & =\left(1-\eta \right){\hat{\alpha }}^{t-1}+\eta \frac{\hat{y}}{{\hat{k}}^{x^{\prime }x^{\prime }}+\lambda }, \end{array}$$

(14) $$\begin{array}{cc} x^{t} & =\left(1-\eta \right)x^{t-1}+\eta x^{\prime }, \end{array}$$

where *η* is a learning rate.

The details of our proposed method are shown in Algorithm 1.
**Algorithm 1** Proposed Tracking Algorithm  1:Initialize the model coefficients $\hat{\alpha }$ and the target appearance x with the bounding box $B_{1}$ given in the 1-st frame.  2:**for** *i* = 2 to end of the sequence **do**  3: Sampling image patches $z^{s}$ in different scale s∈S  4: **for** each scale in $s_{i}^{n}\in S$ **do**  5:  Calculate the response map $R_{i}^{s^{n}}=F^{-1}\left({\hat{k}}^{xz}\cdot \hat{\alpha }\right)$  6:  $p\left(o_{i}|s_{i}^{n}\right)\leftarrow \max\left(R_{i}^{s^{n}}\right)$  7:  $p\left(s_{i}^{n}|o_{i}\right)\leftarrow p\left(o|s_{i}^{n}\right)\times \int p\left(s_{i}^{n}|s_{i-1}\right)p\left(s_{i-1}|o_{i-1}\right)ds_{i-1}$  8: **end** **for**  9: $s^{\prime }=arg\max_{s_{i}^{n}}\left(p\left(s_{i}^{n}\right)\right)$10: calculate the PSR(i) by Equation 12 in the most likely scale $s^{\prime }$11: **if** $PSR\left(i\right)>T_{u}$ **then**12:  update the model coefficient $\hat{\alpha }$ and x13: **end** **if**14: **if** $PSR\left(i\right)>T_{d}$ **then**15:  target position $p_{i}=arg\max\left(R_{i}^{s^{\prime }}\right)$16:  target scale $s_{i}=s^{\prime }$17: **else**18:  target position $p_{i}=p_{i-1}$19:  target scale $s_{i}=s_{i-1}$20: **end** **if**21:**end** **for**

## 5. Experiments

In this section, we firstly describe the experimental details and the value of parameters in our proposed algorithm. Then, we offer a comprehensive evaluation of this algorithm on a large-scale benchmark and compare some state-of-the-art trackers with ours. The results show that our algorithm has high performance for the object tracking problem.

### 5.1. Implementation Details

Firstly, we list some details of our algorithm. Image feature has a significant effect on the performance of the tracking algorithm. In order to increase the robustness of the our tracker, we use an integration feature. We combine the Histogram of Oriented Gradient (HOG) feature and the color names (CN) as the descriptor. The HOG feature that we choose is the compressive HOG used in [21], which is a 31-dimensional vector, with 27 dimensions corresponding to different orientation channels and four dimensions corresponding to overall gradient energy. The color names that we use are mentioned in the research [22], which maps the R-G-B values into 11 linguistic color labels. The color name descriptor is widely used in various modern trackers [23,24] and is verified as a stable color descriptor.

For the scale estimation method, the greater the number of samples of scale variable $s_{t}$, the more accurate the estimation of $s_{t}$. However, as the number of samples increases, the speed of the algorithm will decrease. In order to balance the efficiency and the accuracy, we set the sample number of $s_{t}$ to 15. The scale change of the target between successive frames is slight, so the wide sampling range of $s_{t}$ is useless. By choosing the value of the variance *σ*, we can restrain the major samplings of $s_{t}$ in the range of $0.95s_{t-1}∼1.05s_{t-1}$. Setting $\sigma =0.025s_{t-i}$ ensures this.

We use the Gaussian kernel $k\left(x,x^{\prime }\right)=\exp\left(-\frac{|x-x^{\prime }{|}^{2}}{\sigma^{2}}\right)$ to map the input feature into a non-linear space. The value of the *σ* is 0.2. The learning rate *η* is set to 0.1.

The two thresholds $T_{u}$ and $T_{d}$ are determined by experiments. By testing our algorithm on several tracking videos, we can find that when the target is complete the PSR is larger than 12, the slight occlusion makes the PSR drop to around 9, and the heavy occlusion reduces PSR down to 5. The PSR values in these three cases are almost consistent in every sequence. Therefore, we set the value of $T_{u}$ and $T_{d}$ to 9 and 5, respectively, in our algorithm.

It is worth noticing that we fix all the parameters’ values in all sequences in the TB-50 dataset to ensure the fair comparison to other algorithms. All of the experiments are implemented in MATLAB R2015a on a PC with Intel i7-5930K CPU (3.5 GHz) with 64 GB memory.

### 5.2. Evaluation

In order to evaluate our algorithm, we examine our approach on the TB-50 dataset [16] and three additional challenge sequences, *Bolt2*, *Board* and *Girl2*. There are a total of 52 sequences that are recorded in various scenarios and contain different challenges such as illumination variation, scale variation, occlusion, deformation, etc. We compare our approach with all 29 popular algorithms mentioned in [16], which includes Struck [3], TLD [1], L1APG [18], SCM [2], ASLA [25], CT [13], etc. In order to compare our approach more comprehensively, we add the KCF [4] and the DSST [6] as the comparison. The former is the basis of our algorithm and the latter is the closest algorithm to ours. Two widely used evaluation metrics—distance precision and overlap precision—are given under two test schemes. The two different test schemes are called one-pass evaluation (OPE) and temporal robustness evaluation (TRE). In order to analyze the trackers more completely, the attribute-based evaluation is added.

#### 5.2.1. Quantitative Evaluation

The quantitative comparison of all 32 trackers (our algorithm and other 31 trackers) is given by the distance precision and the overlap precision. The distance precision is based on the center location error, which is the Euclidean distance between the center of the tracking bounding box and the ground truth. The distance precision shows the percentages of frames in which the center location error is less than the given threshold. The overlap precision is based on the PASCAL VOC Overlap Rate (VOR), which is defined by $VOR=\frac{BBox_{t}\cap BBox_{g}}{BBox_{t}\cup BBox_{g}}$, where the $BBox_{g}$ and $BBox_{t}$ mean the ground truth bounding box and the tracking bounding box, respectively. ∩ and ∪ represent the intersection and union of two regions. The overlap precision shows the percentage of the frames in which the VOR surpasses the given threshold.

These two evaluation metrics are measured in two methods, one-pass evaluation (OPE) and the temporal robustness evaluation (TRE). The OPE initializes the tracker with the ground truth location in the first frame, and the tracker runs throughout the entire sequence. In the TRE method, a tracker is evaluated 20 times in a sequence to avoid the sensitivity of the tracker initialization. Each test starts from a particular frame and stops at the end of the sequence.

**Figure 4:**
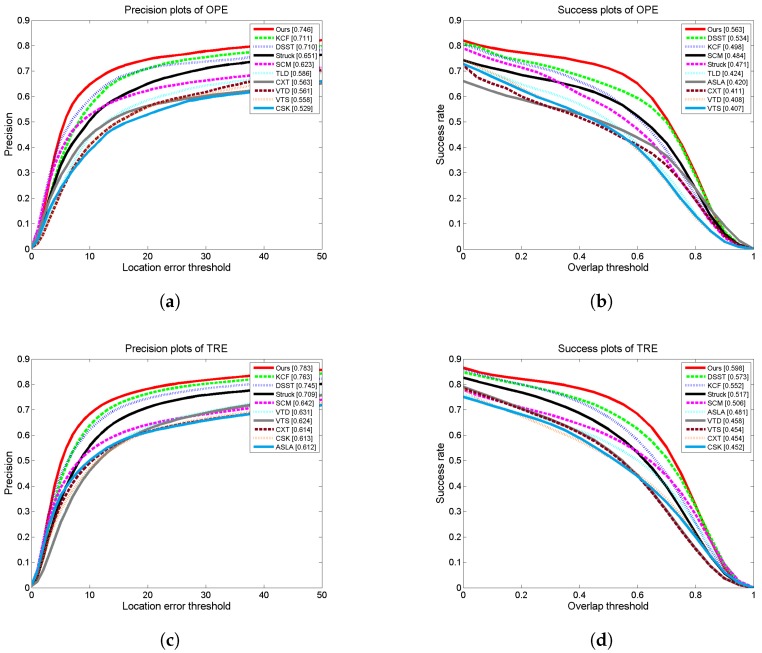
Quantitative results of the top 10 trackers on TB-50. (**a**) Distance precision based on OPE; (**b**) Success rate based on OPE; (**c**) Distance precision based on TRE; (**d**) Success rate based on OPE.

The compared results are shown in Figure 4. These figures show the success rate under a special threshold, which is 0 to 50 pixels for distance precision and 0 to 1 for the overlap precision. We only show the performances of the top 10 trackers among the total 32 trackers. For the distance precision, the trackers are ranked by the success rate at the threshold of 20 pixels. Meanwhile, for the overlap precision, we use the Area Under Curve (AUC) score to rank different trackers.

From Figure 4, we can see that our proposed approach achieves the best performance among the 32 trackers. Figure 4a,b shows the distance precision and the overlap precision in OPE. In this case, our method obtains a 0.746 success rate at a 20-pixel threshold in the distance precision and the AUC score of 0.563 in the overlap precision, respectively. In distance precision, our method improves the performance by 4.9% when compared with the second best tracker KCF. In overlap precision, our method outperforms the DSST, the second-ranked tracker, by 5.4%. Figure 4c,d shows the distance precision and the overlap precision in TRE. For the distance precision, our method just outperforms the KCF by 2% with a success rate of 0.783. The AUC score of our method is 0.598 in the overlap precision plot, and our method surpasses the DSST by 4.4%.

It is worth noting that the ranking results of these trackers are inconsistent under different evaluation metrics. For example, the KCF is the second best tracker when evaluated with distance precision, but it ranks third when evaluated with the overlap precision. It is mainly because different metrics focus on various characteristics of the tracker. The distance precision only focuses on the center location of the target regardless of the size of the target. In contrast to this, the overlap precision, which considers the location and the suitability of the estimation and the ground truth at the same time, is a strict measure, getting a more robust metric result. Nonetheless, our method achieves the best performance for both metrics.

The speeds of different algorithms are also compared. The average frames per second (FPS), which is evaluated for all sequences in TB-50, of the top 10 algorithms in our experiments, are listed in Table 1. It is obvious that KCF, CSK, DSST and ours, which are based on correlation filters, have higher speeds than others. The speeds of our method and DSST are slower than the KCF and CSK because of the additional target scale estimation.

#### 5.2.2. Qualitative Analysis

**Figure 5:**
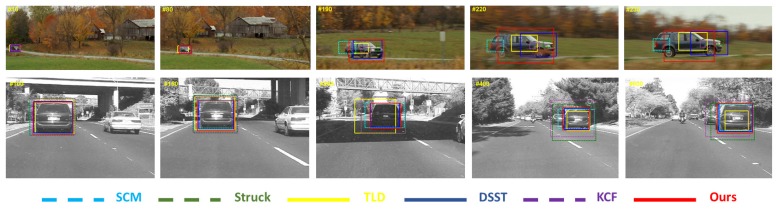
Screenshots of some tracking results in scale variation sequences. These frames are extracted from sequences *carscale* and *car4*, from top to bottom.

**Figure 6:**
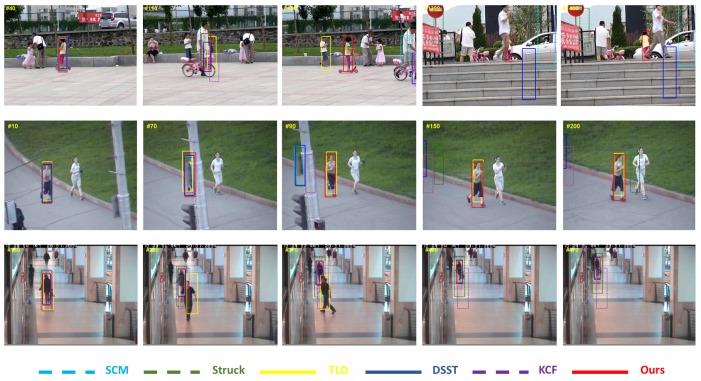
Screenshots of some tracking results in occlusion sequences. These frames are extracted from sequences *girl2*, *jogging* and *walking2*, from top to bottom.

**Figure 7:**
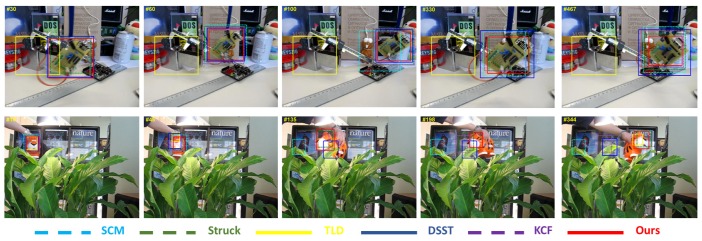
Screenshots of some tracking results in deformation sequences. These frames are extracted from sequences *board* and *tiger2*, from top to bottom.

The tracking results predicted by different trackers are shown in Figure 5, Figure 6 and Figure 7. These figures illustrate the tracking results for several representative sequences, which include almost all of the challenges faced in tracking problems such as illumination variation, scale variation, deformation, occlusion, in-plane rotation, out-of-plane rotation, etc. In order to compare the results clearly and effectively, we only show the results of our algorithm and the other five best trackers ranked by our evaluation.

In Figure 5, we show our results in two scale variation sequences: *ScaleCar* and *Car4*. In the *ScaleCar* sequence, the main challenge is the scale variation accompanied with partial occlusion. The ratio of the maximal target size to the minimal one is more than 30 when the target vehicle approaches the camera from far away. SCM, TLD, DSST and our method can adapt to the target scale change. It is obvious that the four trackers above work well when the scale changes slightly. After the second hundredth frame, SCM, TLD and DSST only can capture part of the car. In other words, they fail to handle the target scale change. In contrast, our method can accurately track the entire car. For the *Car4* sequence, the target undergoes scale variation and illumination changes. In this sequence, our method and DSST work well. Other trackers drift away from the ground truth.

Figure 6 shows tracking results in three sequences, in which the targets are occluded heavily or totally. In the *girl2* sequence, the girl is utterly occluded twice: one happens near the #120 frame, the other happens near the #1300 frame. When the girl is occluded for the first time, our tracker stays in the place where the target disappears thanks to our retained scheme. Other trackers are disturbed by the similar object (the adult near the girl) and drift away with the similar object. Thus, when the target reappears in the scene, our method can re-detect it. It is noticeable that the detection component in TLD can strengthen the tracking result, but it is apt to drift when a similar object appears near the target. This is illuminated in the #190 frame, where TLD re-initializes the tracker on an incorrect object, i.e., the boy near the girl. In the *jogging* sequence, only our method and TLD can track the target reliably under total occlusion. Other algorithms keep the tracking results on the obstruction when the target appears again. In the *walking2* sequence, the target is a walking woman who walks away from the camera and is occluded by another walking man. Our work and DSST can work well. In comparison, TLD tracks the wrong object—the walking man—and can not detect the target when occlusion is over. KCF drifts away due to the target appearance change, when the occlusion occurs.

The tracking results of targets with deformation are shown in Figure 7, which contains two challenge sequences *board* and *tiger2*. In the *board* sequence, the target deformation is caused by the out-of-plane rotation accompanied with a slight scale change. Background clutter is another challenge in this sequence. From Figure 7, the TLD gets failure near the #30 frame in which the target is disturbed by the complicated background. The deformation can be seen by comparing the target in the #60, #100, #330 and #467 frames. The tracking results in these frames show that our algorithm can accurately predict both the location and the scale of the target in the case of deformation. In the *tiger2* sequence, the target undergoes deformation and slight occlusion. The deformation in this sequence is more drastic than that in the *board* sequence. When the deformation is intensive, only our method and Struck can track the target well. SCM, DSST and KCF track the wrong object, while TLD reports that the target is absent in these frames. The ability of our algorithm to deal with deformation benefits from our template update scheme.

#### 5.2.3. Attribute-Based Evaluation and Analysis

**Figure 8:**
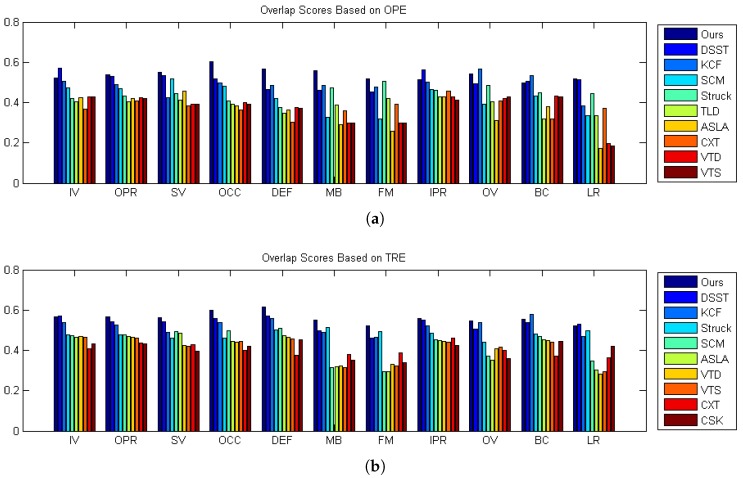
The performance of different attributes. (**a**) Performance scores based on OPE; (**b**) performance scores based on TRE. The meanings of the abbreviations, labeled on the horizontal axis, are list following: IV: illumination variation, SV: scale variation, OCC: occlusion, DEF: deformation, MB: motion blur, FM: fast motion, IPR: in-plane rotation, OPR: out-of-plane rotation, OV: out of view, BC: background clutters and LR: low resolution.

In the benchmark dataset [16], the sequences are annotated with 11 attributes to indicate the types of challenges in the tracking problem. The challenges include illumination variation, scale variation, occlusion, deformation, motion blur, fast motion, in-plane rotation, out-of-plane rotation, out-of-view, background clutters and low resolution. Every sequence includes one or more challenges. In order to make the analysis more complete and clear, we supply the comparison evaluation of these attributes, respectively. In Figure 8, the overlap AUC scores of each tracker in different sequence attributes are shown as a histogram.

From Figure 8, we can conclude that our method outperforms other state-of-the-art trackers for most of the 11 attributes. More specifically, our method is good at dealing with scale variation, occlusion, deformation, in-plane rotation and out-of-view rotation. The reason why our method can handle the target scale variation and occlusion is that we have the scale and occlusion estimation components in our algorithm. In the SV subset, four top-ranked trackers are our method, DSST, SCM and ASLA. All of these trackers are scale adaptive. The SCM and ASLA use particle filters to predict the target state, while DSST and ours contain a specialized scale estimate method in two different ways. The scale variation is so common in tracking that a special component must be designed to handle target scale changes. The deformation, in-plane rotation and out-of-plane rotation can be treated as the target appearance change. Our strict update strategy insures the correctness of the template updating, which can adapt to the target appearance change.

In more detail, scores of the performance for all of the 11 attributes are listed in Table 2. In this table, we show the top 10 trackers’ AUC scores in a column with the same attribute. The red text indicates the corresponding tracker having the first-highest score in the given attribute, blue means the second-highest, and green means the third-highest.

## 6. Conclusions

In this paper, a robust scale adaptive tracking algorithm based on the correlation filter is proposed. We introduce a scale estimate method via Sequence Monte Carlo Framework to cope with the scale variation in tracking. Meanwhile, PSR of the response map is employed to indicate the target completeness, which can handle the occlusion effectively. A strict update strategy is designed to tackle target appearance changes and avoid template degradation. Additionally, the hybrid feature enhances the tracker robustness. Our method evaluates the TB-50 dataset using the OPE and TRE evaluation methods. Both the distance precision and the overlap precision are measured. The experimental results demonstrated that our method outperforms state-of-the-art trackers.

## Figures and Tables

**Table 1:** The speed comparison of different algorithms.

	Ours	KCF	DSST	Struck	SCM	VTD	VTS	CXT	CSK	ASLA
FPS	28.7	161	19.1	15.8	0.39	4.45	4.5	11.9	282.9	6.6

**Table 2:** The overlap scores on attribute based evaluation. (**a**) scores of trackers based on OPE; (**b**) scores of trackers based on TRE. The texts in the red, blue and green color indicate the first, second and third highest score respectively in every column.

(**a**)											
	IV	SV	OC	DEF	MB	FM	IPR	OPR	OV	BC	LR
Ours	0.524	0.539	0.551	0.603	0.567	0.560	0.517	0.514	0.543	0.496	0.519
DSST	0.572	0.528	0.535	0.520	0.467	0.462	0.455	0.563	0.494	0.507	0.514
KCF	0.506	0.489	0.425	0.498	0.486	0.483	0.476	0.500	0.565	0.533	0.384
SCM	0.473	0.468	0.517	0.480	0.420	0.328	0.321	0.463	0.390	0.433	0.333
Struck	0.421	0.432	0.445	0.407	0.378	0.475	0.505	0.463	0.486	0.450	0.444
TLD	0.405	0.403	0.412	0.391	0.346	0.387	0.421	0.429	0.405	0.318	0.335
ASLA	0.426	0.422	0.455	0.382	0.363	0.292	0.258	0.429	0.312	0.379	0.174
CXT	0.369	0.410	0.383	0.365	0.302	0.359	0.390	0.456	0.409	0.320	0.370
VTD	0.428	0.426	0.394	0.399	0.377	0.298	0.297	0.427	0.421	0.431	0.197
VTS	0.429	0.419	0.391	0.394	0.370	0.298	0.298	0.414	0.428	0.428	0.187
(**b**)											
	IV	SV	OC	DEF	MB	FM	IPR	OPR	OV	BC	LR
Ours	0.566	0.567	0.564	0.600	0.614	0.549	0.523	0.560	0.546	0.553	0.521
DSST	0.571	0.542	0.541	0.558	0.571	0.496	0.460	0.550	0.506	0.539	0.530
KCF	0.538	0.525	0.488	0.539	0.559	0.491	0.467	0.523	0.539	0.580	0.470
Struct	0.477	0.478	0.462	0.460	0.501	0.513	0.492	0.486	0.441	0.480	0.497
SCM	0.472	0.479	0.494	0.499	0.507	0.314	0.296	0.453	0.370	0.469	0.378
ASLA	0.466	0.468	0.486	0.446	0.475	0.320	0.292	0.448	0.352	0.452	0.301
VTD	0.470	0.464	0.422	0.442	0.465	0.322	0.331	0.443	0.406	0.449	0.283
VTS	0.466	0.460	0.419	0.444	0.457	0.316	0.323	0.442	0.415	0.439	0.296
CXT	0.409	0.435	0.427	0.401	0.374	0.381	0.387	0.460	0.400	0.373	0.365
CSK	0.430	0.431	0.395	0.419	0.451	0.349	0.337	0.424	0.358	0.445	0.420
